# Regulation of Age-Related Protein Toxicity

**DOI:** 10.3389/fcell.2021.637084

**Published:** 2021-03-05

**Authors:** Anita Pras, Ellen A. A. Nollen

**Affiliations:** Laboratory of Molecular Neurobiology of Ageing, European Research Institute for the Biology of Ageing, University Medical Centre Groningen, University of Groningen, Groningen, Netherlands

**Keywords:** protein homeostasis, protein quality control, aggregation, phase separation, amyloid, aging

## Abstract

Proteome damage plays a major role in aging and age-related neurodegenerative diseases. Under healthy conditions, molecular quality control mechanisms prevent toxic protein misfolding and aggregation. These mechanisms include molecular chaperones for protein folding, spatial compartmentalization for sequestration, and degradation pathways for the removal of harmful proteins. These mechanisms decline with age, resulting in the accumulation of aggregation-prone proteins that are harmful to cells. In the past decades, a variety of fast- and slow-aging model organisms have been used to investigate the biological mechanisms that accelerate or prevent such protein toxicity. In this review, we describe the most important mechanisms that are required for maintaining a healthy proteome. We describe how these mechanisms decline during aging and lead to toxic protein misassembly, aggregation, and amyloid formation. In addition, we discuss how optimized protein homeostasis mechanisms in long-living animals contribute to prolonging their lifespan. This knowledge might help us to develop interventions in the protein homeostasis network that delay aging and age-related pathologies.

## Introduction

Declining protein homeostasis is a major cause of age-related diseases (Koga et al., 2011; López-Otín et al., 2013; Stroo et al., 2017). Tight regulation of protein homeostasis is required to maintain a stable proteome. Regulatory mechanisms include correct protein folding and removal of proteins that are no longer functional or required (Hipp et al., 2019). The ability of the protein homeostasis system to stabilize native proteins declines with age, resulting in protein misassembly, aggregation and cellular toxicity (reviewed in Hipp et al., 2019). Many forms of neurodegenerative diseases are age-dependent and develop in parallel to a decline in protein homeostasis pathways (reviewed in Klaips et al., 2018; Hipp et al., 2019). Recent studies have focused on age-related changes in protein homeostasis and have identified remarkable differences in the protein homeostasis systems of long-living species and their closely related short-living species (Gruber et al., 2015; Rodriguez et al., 2016; Du et al., 2020; Lagunas-Rangel, 2020). Although most protein homeostasis pathways are generally the same, differences in expression and function of certain protein homeostasis components may contribute to longevity and healthy aging.

Most of what we know about protein homeostasis and aging has come from studies in *Caenorhabditis elegans*, *Saccharomyces cerevisiae*, *Drosophila melanogaster*, and *Mus musculus*. These models are useful for studying aging and age-related diseases because they are easy to maintain and have short lifespans (i.e., they age quickly) (Valenzano et al., 2017). In addition, they are well characterized and their genomes have been fully sequenced (C. elegans Sequencing Consortium, 1998; Adams et al., 2000; Waterston et al., 2002; Engel et al., 2014). Since a decline in protein homeostasis has been proposed to cause aging, researchers have looked for ways to optimize protein homeostasis to prevent or delay the development of age-related diseases. Long-living *C. elegans*, *S. cerevisiae*, *D. melanogaster* and *M. musculus* mutant models help us understand which mechanisms are important for longevity (Kenyon et al., 1993; Clancy et al., 2001; Brown-Borg and Bartke, 2012; Muid et al., 2019). However, examining those species that have naturally evolved as long-living might provide additional clues about the mechanisms of healthy aging (Cohen, 2018). For example, long-living animal species like bivalve mollusks, naked mole-rats, and bats have developed mechanisms to reduce reactive oxygen species (ROS) production and to protect their proteomes from unfolding (Brunet-Rossinni, 2004; Gruber et al., 2015; Treaster et al., 2015). Increased chaperone production, autophagy, and proteasome activity further prolong the lifespan of these long-living species (Pérez et al., 2009; Rodriguez et al., 2014, 2016).

In this review, we discuss the most important protein homeostasis mechanisms for a healthy proteome. We summarize the current knowledge on factors and pathways that play a role in mammalian protein homeostasis and how changes in protein homeostasis can contribute to aging. We also discuss what we can learn from protein homeostasis machineries in short- and long-living animal species. These lessons could suggest interventions for improving protein homeostasis in humans to prevent or delay the onset of age-related diseases.

## Protein Homeostasis Declines With Aging

### Protein Synthesis

The protein homeostasis system regulates protein function from the moment a protein is synthesized to when it is degraded or secreted. A strict balance between protein synthesis, folding, and degradation is needed to maintain the protein levels required for normal cellular function without overwhelming the protein quality control machinery. The number of proteins that can be synthesized depends on several factors, including the availability of mRNA transcripts and ribosomes for protein translation (Walther et al., 2015; Hipp et al., 2019). The presence and activity of translation initiation (eIF) and elongation factors (eEF) (e.g., eIF2α, eIF4E and eEF2) additionally determine the rate of protein synthesis (Papadopoli et al., 2019; Xie et al., 2019; Anisimova et al., 2020). For example, phosphorylation of eIF2α inhibits protein synthesis, and multiple studies have shown that a reduction in protein translation improves health and extends lifespan (Hansen et al., 2007; Pan et al., 2007; Pakos-Zebrucka et al., 2016; Xie et al., 2019). Other initiation and elongation factors are regulated by the mechanistic target of rapamycin complex 1 (mTORC1)-signaling pathway (Papadopoli et al., 2019; Xie et al., 2019). mTORC1 is an important regulator of protein synthesis and mediates, for example, the phosphorylation eIF4E-binding proteins (4E-BPs) that control the activity of the translation initiation factor eIF4E (Papadopoli et al., 2019). In addition, mTORC1 mediates translation accuracy by controlling eEF2 kinase (Xie et al., 2019).

During aging, protein synthesis rates decline (Dhondt et al., 2017; Yang et al., 2019). Inhibition of protein translation could be a protective mechanism to reduce the burden on protein quality control machineries and to restore protein homeostasis (Hipp et al., 2019). This decline may in part result from a reduction in ribosome abundance during aging (Walther et al., 2015). On the other hand, however, mTOR activity increases with increasing age, which results in increased protein synthesis rates, and reduced expression of chaperones, autophagy and proteasome activity (Yang et al., 2012; Papadopoli et al., 2019). With increasing age, imbalances in protein homeostasis can therefore lead to chronic stress conditions, reduced phosphorylation and lack of inhibition of factors involved in protein synthesis (Ben-Zvi et al., 2009; Taylor, 2016).

### Molecular Chaperones

**FIGURE 1:**
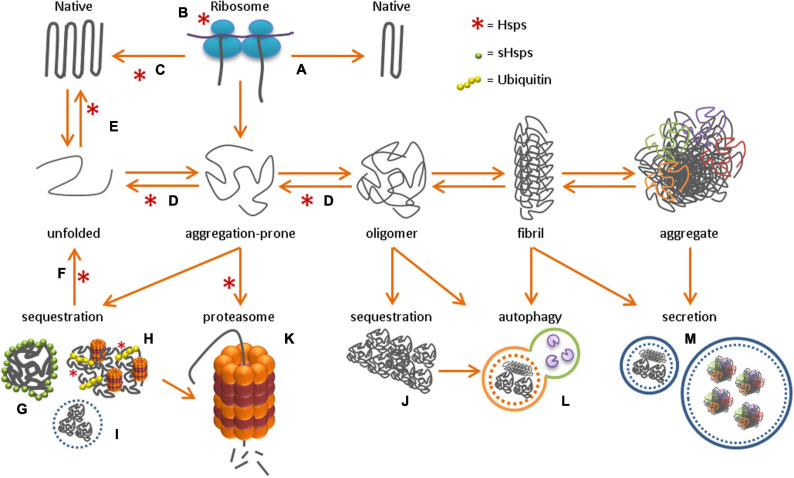
Protein homeostasis network After the synthesis of a new polypeptide chain, the folding of a protein toward its native conformation can either directly occur through intermolecular interactions **(A)**, or with the help of molecular chaperones. Chaperones can either promote correct folding co-translationally at the ribosome **(B)** or post-translationally in the cytosol **(C)**. Upon toxic stress conditions, protein misfolding toward aggregation-prone species and oligomers can occur, followed by fibril formation and the formation of insoluble protein aggregates. To protect the cell from these toxic protein species, active unfolding **(D)**, re-folding **(E)** and re-solubilization of aggregates through disaggregation **(F)** occurs, with the help of molecular chaperones, while sHsps perform their function as ‘holdase’ in sHSP oligomers **(G)**. Alternatively, misfolded protein species can be sequestered in, for example, the JUNQ **(H)**, nucleolus **(I)** or aggresome **(J)**. Alternatively, proteins can be degraded by the proteasome **(K)** or through autophagy **(L)**, or secreted into the extracellular environment **(M)**.

After a polypeptide chain is synthesized, it undergoes structural conversions before being folded into its stable native state (Hartl, 2017). Many proteins need this stable 3D-structure to function properly, and correct folding is largely determined by the amino acid sequence of the protein (Anfinsen, 1973). Intra-molecular amino acid interactions like hydrogen bonds, disulfide bonds, electrostatic interactions, and hydrophobic interactions guide proteins toward their native conformation (Longo and Blaber, 2016). These intra-molecular forces are usually sufficient to fold short polypeptide chains, but ATP-dependent molecular chaperones are needed to fold larger globular proteins into their native conformation (Figure 1).

The main molecular chaperone families that are involved in *de novo* folding of newly synthesized proteins are the heat-shock proteins (Hsps) 70 and 90, and chaperonins. The most universal chaperone is Hsp70, which has multiple functions in protein homeostasis, and is involved in nascent protein folding at the ribosome, in post-translational refolding of aggregation-prone proteins in the cytosol, and in re-solubilization of aggregates (Figure 1) (reviewed in Matthias P Mayer and Gierasch, 2019). Once bound to Hsp70, substrate proteins are stabilized and ready for folding or refolding with the help of co-chaperones (e.g., Hsp40, Hsp90) or chaperonins (Hsp60) (Kim et al., 2013; Genest et al., 2015, 2019). ATP-dependent chaperones protect against protein aggregation by promoting the correct folding of unfolded or aggregation-prone proteins. In contrast, small heat shock proteins (sHsps) are ATP-independent chaperones that bind and hold onto unfolded protein species (reviewed in Mogk et al., 2018). During cellular stress, sHsps keep unfolded proteins in a refolding-competent state so that refolding can be initiated upon stress relief (Cashikar et al., 2005; Escusa-Toret et al., 2013; Ungelenk et al., 2016; Shen et al., 2019). sHsps cannot refold aggregation-prone proteins to their native state. For refolding, they require the assistance of ATP-dependent chaperones, like Hsp70. Under proteotoxic stress conditions chaperones levels are increased by cellular stress response pathways, such as the heat shock response. This cytosolic stress response, is regulated by heat shock factors (HSFs), of which HSF1 is the best characterized (Chaudhury et al., 2021).

Chaperones are also important when the number of misfolded proteins increase with aging. However, studies in C. elegans showed that the ability to activate the heat shock response reduces with increasing age (Ben-Zvi et al., 2009; Shemesh et al., 2013; Brehme et al., 2014; Labbadia and Morimoto, 2015). This decline in ability occurs early in adulthood, at the onset of oocyte biomass production, and seems a consequences of a reduced expression of the H3K27 demethylase jmjd-3.1 (Shemesh et al., 2013; Labbadia and Morimoto, 2015). A decline in the ability to induce chaperone expression with increasing age, was also found in senescent human lung fibroblasts (Sabath et al., 2020). In contrast, Walther and colleagues did not observe major changes in Hsp70 and Hsp90 expression in C. elegans throughout life, while sHsp expression even increased dramatically during aging (Walther et al., 2015). Most sHsps accumulated in aggregates during aging, indicating that cells actively sequester their proteins to cope with the increase in aggregation-prone proteins (Walther et al., 2015). In addition, the number of sHsp-associated inclusions strongly increased with aging in the long-living daf-2 C. elegans mutant, which suggests that sequestration is a protective mechanism (Walther et al., 2015). These findings agree with previous results in human tissue samples, where increased expression of sHsp genes was observed in the aging brain, and elevated sHsp levels were detected in skeletal muscles of aged individuals (Yamaguchi et al., 2007; Brehme et al., 2014).

### Unfolded Protein Responses in the ER

Another important stress response pathway that regulates protein-folding is the unfolded protein response in the endoplasmic reticulum (ER), the UPR^ER^ pathway (Taylor, 2016; Frakes and Dillin, 2017). The ER creates a tightly regulated environment for folding, processing, and secretion of newly synthesized secretory and membrane proteins. The ER detects and responds to any imbalances in protein homeostasis, such as hypoxia, nutrient deprivation and excessive protein oxidation (Martínez et al., 2017). The three most important factors responsible for the UPR^ER^ are the transmembrane sensors inositol-requiring protein 1 (IRE1), activating transcription factor 6 (ATF6), and PKR-like ER kinase (PERK) (Frakes and Dillin, 2017). During non-stressful physiological conditions, these sensors are quiescent through interaction with the ER chaperone binding immunoglobulin protein (BiP). When the number of unfolded or aggregation-prone proteins increases, BiP is recruited and titrated away from the stress sensors, which activates the UPR^ER^ (Bertolotti et al., 2000; Carrara et al., 2015). In addition, unfolded or aggregation-prone proteins can directly bind to the UPR^ER^ sensors and activate the UPR^ER^ (Gardner and Walter, 2011).

Activation of different UPR^ER^ components has been associated with lifespan extension, and results in reduced protein synthesis and increased expression of chaperones and factors contributing to proteasomal degradation (Taylor and Dillin, 2013; Luis et al., 2016; Martínez et al., 2017). Upon UPR^ER^ activation, the transcription factor X-box binding protein 1 (Xbp1) promotes transcription of genes encoding for chaperones and factors that promote ER-associated degradation. In neurons, overexpression of Xbp1 can prevent the decline in ability to induce the UPR^ER^ with aging (Frakes et al., 2020). At the same time, enhanced lipid biogenesis increases protein folding and protein degradation in the ER (Chalmers et al., 2017; Frakes and Dillin, 2017). However, the ability to induce the UPR^ER^ and its downstream targets decline with increasing age (Sabath et al., 2020; Taylor and Hetz, 2020). In addition, absolute UPR^ER^-induced chaperone levels decrease during aging and the UPR^ER^-regulated chaperones that are still present, accumulate increasing amounts of oxidative damage (Taylor, 2016). The role of UPR^ER^ in aging has recently been extensively reviewed by Taylor and Hetz (Taylor and Hetz, 2020).

### Unfolded Protein Responses in the Mitochondria

Another important unfolded protein response mechanism is the mitochondrial UPR (UPR^mt^). The UPR^mt^ is activated in response to different kinds of stressors. Examples include excessive amounts of reactive oxygen species (ROS) or impaired import of mitochondrial proteins due to damaged protein accumulation (Nargund et al., 2012; Fiorese et al., 2016; Shpilka and Haynes, 2018). Activation of the UPR^mt^ is required for repair of the mitochondrial network and maintenance of the mitochondrial function for the cell (Shpilka and Haynes, 2018). Mild, temporary mitochondrial stress is beneficial as it maintains protein homeostasis through upregulation of HSF-1 (Labbadia et al., 2017).

Chronic activation of the UPR^mt^, however, which also occurs during aging, gradually impairs mitochondrial ATP production, and increases electron leakage (Shpilka and Haynes, 2018). Aging has therefore been associated with increased levels of ROS. A chronic increase in ROS production causes oxidative stress and contributes to the accumulation of DNA damage (e.g., mutations and chromosomal aneuploidies), RNA damage, and further mitochondrial damage (Korovila et al., 2017). All of which contribute to the increase in aggregation-prone proteins with aging (Faggioli et al., 2012; Forsberg et al., 2012; Liu et al., 2020). Furthermore, proteins can be directly damaged by oxidation, which induces structural changes and makes proteins more aggregation-prone (Serebryany et al., 2016; Lévy et al., 2019). Especially the amino acid cysteine (Cys) is susceptible due to the presence of a nucleophilic thiol-group, but also the amino acids tryptophan, tyrosine, methionine and histidine are prone for oxidation (Lévy et al., 2019). In addition, oxidation can alter the side-chain charges of amino acids, which affects native folding and repulsion between proteins (known as colloidal stability) (Samantha S. Strickler et al., 2006; Gribenko and Makhatadze, 2007; Beerten et al., 2012; De Baets et al., 2014). Altogether, age-related chronic mitochondrial stress results in the accumulation of ROS and damaged proteins, and in a reduction in ATP, which further contributes to the decline in mitochondrial function and results in an imbalance in protein homeostasis (Korovila et al., 2017; Shpilka and Haynes, 2018).

### Regulated Sequestration and Disaggregation

Aggregation-prone proteins or prematurely terminated proteins (defective ribosomal products) can be stored in compartments such as the juxtanuclear quality control compartment (JUNQ) or membraneless nuclear bodies (Kaganovich et al., 2008; Mediani et al., 2019). In addition, functional amyloids assemble into storage sites termed amyloid (A)-bodies, to store proteins under stressful conditions. The formation of these storage sites might be a physiological mechanism to immobilize proteins and allow the cell to become dormant (Audas et al., 2016). Once the stressor is released, proteins in the A-bodies disaggregate back to a soluble state with the help of molecular chaperones. Several specialized protein quality control sites have been identified in the mammalian cell (reviewed in Sontag et al., 2017), including the JUNQ, the perivacuolar compartment (aggresome, equivalent to the insoluble protein deposit, IPOD, in yeast), and the nucleolus (Kaganovich et al., 2008; Frottin et al., 2019). Sorting of aggregation-prone cytosolic proteins to these distinct compartments depends on chaperone binding and ubiquitination. Soluble aggregation-prone proteins that are recognized by the protein quality control machinery are ubiquitinated and subsequently transported to the JUNQ (Figure 1; Sontag et al., 2017). The JUNQ contains disaggregating chaperones and 26S proteasomes, which increase the efficiency for refolding or degradation of aggregation-prone proteins (Kaganovich et al., 2008). However, if the quality control machinery is overwhelmed or impaired, aggregation-prone proteins might continue to accumulate. These bigger assemblies are usually directed to aggresomes, which terminally sequester small protein aggregates, including amyloidogenic aggregates (Johnston et al., 1998; Figure 1).

The role of the above-mentioned sequestration compartments in aging remains unclear. Studies indicate that soluble aggregation-prone proteins and oligomers have a pathological role in neurodegenerative diseases like Alzheimer’s disease and Huntington’s disease (Mucke et al., 2000; Arrasate et al., 2004; Iulita et al., 2014). The aggregation of these soluble proteins was therefore suggested as a protective mechanism to prevent cytotoxicity (Arrasate et al., 2004; Cohen et al., 2006; Hong et al., 2014). Indeed, hyperaggregation of amyloid-beta was associated with delayed age-related proteotoxicity of soluble amyloid-beta, upon reduction of insulin/insulin growth factor (IGF) signaling in an Alzheimer’s mouse model (Cohen et al., 2009). As mentioned before, also the upregulation of sHsp inclusions has been associated with lifespan extension in worms (Walther et al., 2015). However, how the formation of storage compartments affect aging remains unclear. While sequestration of aggregation-prone proteins might seem beneficial initially, these temporary storage sites might become permanent insoluble aggregates during chronic stress conditions such as aging. Cells may not be able to tolerate these large aggregates as they might sequester functional proteins, release aggregation-prone species back to the cellular environment, or interfere with cellular processes (Morley et al., 2002; Olzscha et al., 2011; Mogk et al., 2018).

### Phase Separation and Liquid Droplet Formation

**FIGURE 2:**
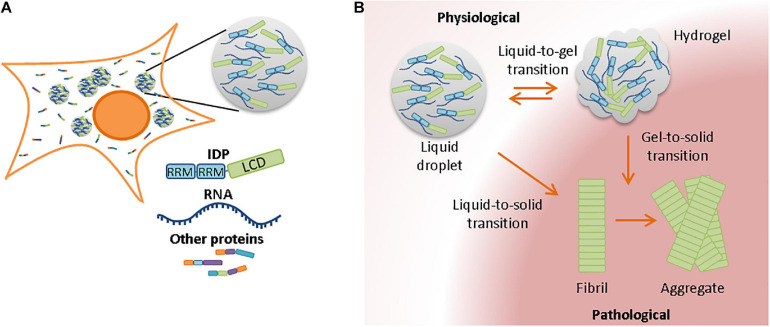
Membrane-less compartments in the cell and their involvement in aggregation with aging **(A)** Liquid-liquid phase separation requires often, but not always, the presence of RNA and RNA-binding proteins. RNA-binding proteins contain RNA-recognition motives and a low-complexity domain in their protein sequences. **(B)** Due to the high concentration of proteins in liquid droplets, a strict protein homeostasis is required to prevent phase transitions from liquid-like compartments to less dynamic states. Although transition from hydrogel to liquid droplet is in some cases still possible, liquid-to-solid and gel-to-solid transitions normally result in the formation of pathological fibrils and insoluble aggregates.

Proteins can also be compartmentalized by membraneless liquid-like organelles, which regulate cellular processes rather than store aggregation-prone and prematurely terminated proteins (Shin et al., 2017; Mediani et al., 2019). Well-known examples of membraneless compartments are P-bodies and stress granules in the cytoplasm, or Cajal bodies in the nucleus. They are normally characterized by their spherical composition and dynamic properties, and are therefore also known as liquid droplets (Brangwynne et al., 2009). These liquid-like compartments are formed by liquid-liquid phase separation (LLPS). Through LLPS, a compartment with a higher molecular concentration than its surrounding is formed. LLPS can be regulated by distinct proteins, including multivalent proteins (Li et al., 2012) and intrinsically disordered proteins (Kato et al., 2012; Uversky, 2017). Intrinsically disordered proteins (also known as natively unfolded proteins) have an amino acid sequence that does not favor folding into a 3D structure by itself. Most, but not all unfolded proteins go through a folding-upon-binding transition as soon as the protein binds to its physiological ligand (Bonetti et al., 2018; Fuxreiter, 2018). While low complexity domains are required for LLPS, interactions between RNA and RNA-recognition motifs further contribute to the assembly of liquid-like droplets (Figure 2A) (Teixeira et al., 2005; Molliex et al., 2015; Zhang et al., 2015). Liquid-like compartments have been implicated in several cellular processes, including organization and regulation of proteins in the cytosol, and the controlled release of sequestered molecules from cellular compartments (Boisvert et al., 2007; Kroschwald et al., 2015; Molliex et al., 2015; Wheeler et al., 2016; Banani et al., 2017; Shin and Brangwynne, 2017). Compartments with regulatory functions include the nucleolus and stress granules. In the event of stress, they regulate signaling molecules, mRNA, and transcription/translation complexes to prevent off-target interactions with other molecules. In addition, studies have suggested a role for liquid droplet formation in the nucleation and polymerization of actin and tubulin bundles (Banjade and Rosen, 2014; Hernández-Vega et al., 2017).

The dynamics of membrane-less compartments with high concentrations of proteins need to be tightly regulated and efficient protein quality control usually prevents the transition of stress granules toward a more solid state (Lechler et al., 2017). However, such phase transitions can occur if protein quality control mechanisms are impaired during aging, which can reduce the dynamic properties of liquid-like compartments and turn them into solid structures (Seguin et al., 2014). Also, the number of stress granules that are formed increases with aging (Lechler et al., 2017). Liquid-to-solid phase transitions occur more often with increasing age, potentially due to the presence of increased amounts of damaged proteins that may be recruited to membrane-less compartments and seed the formation of aggregates (Lechler et al., 2017; Hipp et al., 2019). Stress granules and other membrane-less compartments are enriched in RNA-binding and disordered proteins. Although there are fewer aggregation-prone regions in disordered proteins, their aggregation has been associated with aging and age-related (neurodegenerative) diseases (Linding et al., 2004). Changes in pH, protein concentration, salt concentration, or temperature can affect the viscosity of granules and transform liquid droplets into hydrogels or insoluble amyloid aggregates (Figure 2B; Alberti and Hyman, 2016; Mateju et al., 2017; Peskett et al., 2018). Also mutations in or adjacent to the low complexity domain of RNA-binding proteins could change the biophysical properties of liquid droplets and accelerate liquid-to-solid phase transitions (Patel et al., 2015; Gopal et al., 2017). Stress granules could therefore act as nucleation sites for pathological aggregates. Several neurodegenerative diseases have been associated with the aggregation of RNA-binding proteins, including TAR DNA binding protein of 43 kDa (TDP-43) and fused in sarcoma (FUS) (Patel et al., 2015; Gopal et al., 2017; Peskett et al., 2018; Ray et al., 2020).

### Cellular Factors That Enhance Protein Aggregation and Toxicity

Protein homeostasis prevents toxic formation of protein aggregates. In recent years, however, a range of factors have been identified that can promote misfolding and aggregation of various disease-related proteins. For example, cytoplasmic polyphosphate (polyP) polymers, glucosaminoglycans like heparin, nucleic acids, and metal cations have been found to modify the aggregation of Tau, alpha-synuclein, prion protein, and amyloid-beta, respectively (Uversky et al., 2001; Jeffrey A. Cohlberg et al., 2002; Nandi et al., 2002; Yugay et al., 2016; Wickramasinghe et al., 2019). Although the exact mechanisms remain unresolved, studies suggest that interaction of polyP with Tau and metal cations with alpha-synuclein could alter the native protein conformation, increasing the probability of disease-related aggregation (Uversky et al., 2001; Wickramasinghe et al., 2019).

A group of proteins which were described for their aggregation promoting effects, called Modifiers of Aggregation (MOAGs), were originally identified in a chemical mutagenesis screen in a *C. elegans* model of neurodegenerative diseases. In this screen, MOAG-2 and MOAG-4 promoted polyglutamine aggregation (Van Ham et al., 2010; Sin et al., 2017). MOAG-2 (also known as *lin-26*-related gene 3 [*lir-3*]) was first identified as an aggregation-promoting factor that catalyzed the sequestration and toxicity of polyglutamine in large insoluble aggregates (Sin et al., 2017). However, this sequestration mechanism seemed to be a consequence of MOAG-2/LIR-3 mislocalization from the nucleus to the cytosol in the presence of polyglutamine, rather than a protective compartmentalization mechanism in the presence of aggregation-prone proteins. In the absence of polyglutamine in wild-type *C. elegans*, MOAG-2/LIR-3 regulated the transcription of small non-coding RNAs (Sin et al., 2017).

*Moag-4* encodes a small protein with unknown function that is evolutionarily highly conserved. MOAG-4 is a highly dynamic, intrinsically disordered protein that forms transient alpha-helical secondary structures but not tertiary conformations (Yoshimura et al., 2017). It was shown to act cell-autonomously and independent from quality control mechanisms such as chaperones or proteasomes (Van Ham et al., 2010). Also, its human orthologs, small EDRK-rich factors (SERF)1a and SERF2 were found to enhance off-pathway structural conversions for several unrelated amyloidogenic proteins (Van Ham et al., 2010; Falsone et al., 2012). MOAG-4, SERF1a, and SERF2 promote aggregation of proteins into compact aggregation intermediates that eventually become large, insoluble aggregates. SERF promotes aggregation through direct and transient interactions. Electrostatic interactions between MOAG-4/SERF1a and alpha-synuclein accelerate the formation of alpha-synuclein fibrils (Falsone et al., 2012; Yoshimura et al., 2017; Merle et al., 2019). This aggregation-promoting effect of SERF on alpha-synuclein was recently suggested to be a toxic side effect, mediated by an interaction between SERF1a and the negatively charged C-terminus of alpha-synuclein (Meyer et al., 2019). This study suggested that SERF1a acts as an RNA chaperone in the formation of liquid-like RNA organelles. Under stressful conditions, RNA and alpha-synuclein may compete for SERF binding, possibly favoring an interaction between alpha-synuclein and SERF that accelerates amyloid formation (Meyer et al., 2019). Such stressful conditions could arise during aging as protein homeostasis declines. Under these conditions, SERF-like proteins might turn into toxic factors and threaten the proteome. Nevertheless, the exact function of SERF in the proteome remains elusive.

### Protein Degradation

Numerous mechanisms in the cell regulate protein degradation and secretion. These mechanisms are necessary to maintain physiological protein concentrations, clear the cell from proteins that are no longer required, and to avoid toxic accumulation of non-native proteins. Proteins are eliminated via three main pathways: proteasomal degradation, autophagy, and extracellular secretion (Figure 1).

Proteasome-mediated degradation is regulated by the ubiquitin-proteasome system (UPS), which is the primary route for eliminating non-native monomeric proteins. The autophagy-lysosome pathway (ALP) removes larger proteins, aggregates, or dysfunctional organelles (reviewed in Dikic, 2017). Proteins are directed to the UPS and ALP by chaperones and additional co-factors with ubiquitin ligase activity. The proteasome regulatory particle specifically recognizes ubiquitinated substrates and directs them to the proteasome core particle for degradation (Lander et al., 2012). With the help of ATP-dependent chaperones, the substrate proteins are unfolded and digested into peptides of 2–24 residues by protease enzymes.

The best characterized type of autophagy is macroautophagy, which is the degradation pathway for large components like cellular organelles and protein aggregates. Macroautophagy is mediated by a large family of autophagy-related proteins (ATG proteins) (Noda and Inagaki, 2015). Upon encapsulation of the substrate material, the autophagosome is formed and transported along microtubules to fuse together with lysosomes and form an autolysosome (Yu et al., 2018; Figure 1). Protease enzymes in the lysosome then degrade the encapsulated material. Another autophagy pathway, known as endosomal microautophagy in mammals, involves the bulk or selective degradation of cytosolic proteins by endosomes (reviewed in Tekirdag and Cuervo, 2018). For bulk degradation, cytosolic substrates are directly trapped in late endosomes. For selective degradation, the heat shock cognate 70 (Hsc70) protein is required (Chiang and Dice, 1988; Sahu et al., 2011). Another type of chaperone-mediated autophagy in mammals involves the direct targeting of substrate proteins to the lysosome by Hsc70. Hsc70 regulates the delivery of the substrate protein to the lysosome by interacting with the lysosome-associated membrane protein 2A (Cuervo and Dice, 1996; Salvador et al., 2000). The substrate protein is then unfolded and translocated to the lysosome for degradation.

Protein degradation relieves the cell from protein overload and provides amino acids for further protein synthesis (Suraweera et al., 2012; Dikic, 2017). These processes are important for maintaining a healthy proteome. In *C. elegans*, an age-related decline in proteasomal function and autophagy was observed (Cuervo and Dice, 2000; Paisán-Ruíz et al., 2004; Tonoki et al., 2009; Brehme et al., 2014; Martinez-Lopez et al., 2015; Cho et al., 2018). The imbalance between production and clearance of misfolded proteins correlates with aging and ultimately results in protein supersaturation and aggregation (Ciryam et al., 2013, 2015). The proteostasis network tries to restore these imbalances by upregulating components of the ubiquitin proteasome system (Chondrogianni et al., 2015; Walther et al., 2015). This proteasomal upregulation has been associated with an increased lifespan in *C. elegans* (Chondrogianni et al., 2015).

## Protein Homeostasis in Short- and Long-Living Animal Species

**FIGURE 3:**
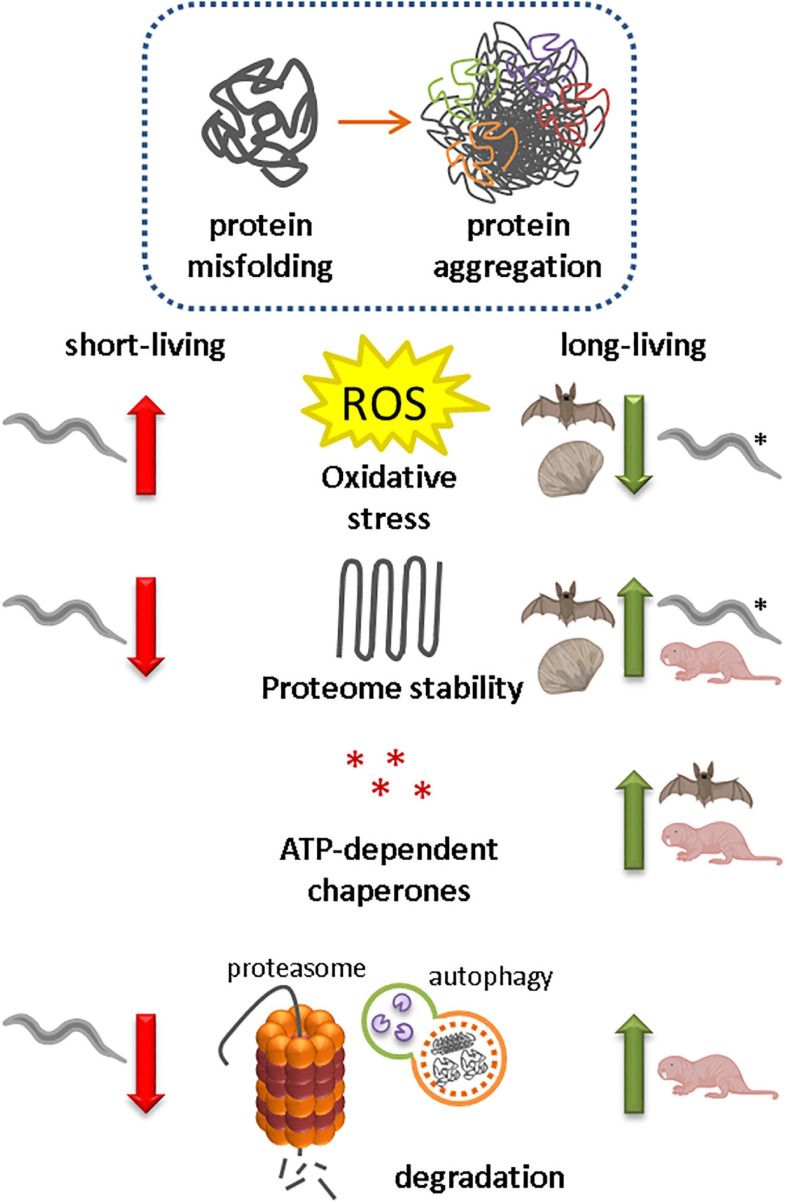
Protein homeostasis capacity in animal species correlates with lifespan A decline in protein homeostasis due to increased oxidative stress, reduced proteome stability, reduced expression and activity of ATP-dependent chaperones and reduced protein degradation, has been associated with protein misfolding and the formation of insoluble protein aggregates. Naturally evolved long-living animal species (bivalve mollusk *Arctica islandica*, some bat species and naked mole-rats) have reduced their ROS production, increased their proteome stability and/or optimized protein folding and degradation pathways for a longer and healthier life. Also long-living *daf-2* mutant *Caenorhabditis elegans* models (*) have lower oxidative stress levels.

Several interventions have been shown to promote health and extend lifespan in model organisms, including upregulation and overexpression of different protein homeostasis components, such as HSF1, Hsp16, 19S proteasomal subunits, and selective autophagy receptors (Hsu et al., 2003; Walker and Lithgow, 2003; Morley and Morimoto, 2004; Vilchez et al., 2012b; Kumsta et al., 2019) (Figure 3). Most of this research was conducted in *C. elegans*, yeast and mouse models, however, understanding how improved protein homeostasis mechanisms contribute to the long lifespans of naturally evolved long-living animal species might additionally help us understand the mechanisms that are important in healthy aging.

### Oxidative Stress Defense and Proteome Protection Contribute to Lifespan

Levels of oxidative stress and antioxidants have been correlated with life-expectancy in the ‘oxidative stress hypothesis of aging’ (Harman, 1956; Csiszar et al., 2007; Shi et al., 2013). In accordance with this hypothesis, long-living *daf-2* mutant *C. elegans* models maintain lower oxidative stress levels during their transition to adulthood than short-living *daf-16* mutant strains do (Knoefler et al., 2012). ROS levels have also been correlated with lifespan in several long-living animals, including the bivalve mollusk species *Arctica islandica*, and the little brown bat species *Myotis lucifugus* (Brunet-Rossinni, 2004; Ungvari et al., 2011, 2013; Gruber et al., 2015). In both animal species, ROS production is low (Brunet-Rossinni, 2004; Ungvari et al., 2011; Gruber et al., 2015). *A. islandica* is an ocean quahog that can live for over 500 years, and the age can be determined by counting the annual growth rings in the shell. In addition to low ROS production, *A. islandica* has shown increased resistance to (mitochondrial) oxidative stressors and most genotoxic stressors compared with shorter-living bivalve species (Salmon et al., 2009; Ungvari et al., 2011, 2013). Especially remarkable is the low level of antioxidant response upon acute stress exposure (Ungvari et al., 2011). In *C. elegans* the antioxidant enzyme superoxide dismutase (SOD) is not required for lifespan regulation, although it is necessary to be able to cope with acute stressors (Van Raamsdonk and Hekimi, 2009, 2012). In *A. islandica*, the exact mechanisms for the remarkable resistance against oxidative stressors remains unknown, but the resistance to genotoxic stressors indicates optimal defense pathways such as strong DNA repair mechanisms, as has also been proposed in several long-living mammals, including some long-living mouse species, muroid rodents, bats and primates (Salmon et al., 2008; Ungvari et al., 2008, 2013; A. Podlutsky et al., 2008). Low ROS levels and resistance to genome damage likely reduce protein damage, but to what extent this contributes to the exceptional long lifespan of *A. islandica* remains to be determined.

In *C. elegans*, significant changes in relative proteins abundance and solubility of the proteome have been reported with increasing age (David et al., 2010; Reis-Rodrigues et al., 2012; Walther et al., 2015). An imbalance between the production and clearance of insoluble proteins correlates positively with aging and ultimately results in protein supersaturation and subsequent protein aggregation (Ciryam et al., 2013, 2015). The formation of insoluble protein aggregates in turn further promotes aging (Reis-Rodrigues et al., 2012; Huang et al., 2019). *A. islandica* has an improved ability to protect its proteome for unfolding stressors (Treaster et al., 2014, 2015; Gruber et al., 2015). Although the optimal living temperature for *A. islandica* ranges between 5 and 15°C, most of its proteins stayed soluble even under extreme temperatures of 100°C (Treaster et al., 2014). In addition, *A. islandica* maintained 45% of its GAPDH activity in muscle tissue in the presence of 6M urea, indicating superior proteome protection (Treaster et al., 2015). The strong resistance to protein unfolding stressors in *A. islandica* could indicate a prominent role for molecular chaperones in stabilizing protein structures. However, ATP-dependent chaperones and small heat shock proteins could not be identified as responsible factors for the effective protein homeostasis in *A. islandica* in this study (Treaster et al., 2015). Which factors exactly are responsible for the stabilization of the proteome in *A. islandica* remain elusive.

### Unique Mechanisms Promote the Health and Lifespan of Naked Mole-Rats

In contrast to *A. Islandica* and *M. lucifugus*, oxidative stress levels are higher in naked mole-rats in comparison with physiologically age-matched mice (Andziak et al., 2005, 2006; Pérez et al., 2009). Naked mole-rats can live for more than 30 years; they have negligible senescence and are resistant to cancer and other age-related diseases (Buffenstein, 2008; Edrey et al., 2011, 2013; Azpurua et al., 2013; Tian et al., 2013). Surprisingly, despite their elevated oxidative stress levels, activities of Cu/Zn superoxide dismutase, Mn superoxide dismutase, catalase and cellular glutathione peroxidase are not higher in naked mole-rats than in mice, and levels do not change with aging (Andziak et al., 2005, 2006). This has also been observed in certain long-living bird and bat species, and contradicts the oxidative stress hypothesis of aging (Andziak et al., 2006; Munshi-South and Wilkinson, 2010). High oxidative stress levels are expected to increase damage to DNA and lipids, and increase production of misfolded proteins. Indeed, damage to DNA, lipids, and proteins was higher in naked mole-rats than in mice (Andziak et al., 2006). The lack of elevated antioxidant levels indicates that other mechanisms are responsible for the long lifespan and resistance to aging in these animals. A recent study indicates the contribution of elevated expression of peroxiredoxin 1 (PRDX1) and thioredoxin reductase 1 (TXNRD1) in the liver of naked mole-rats to their long lifespan (Heinze et al., 2018). *PRDX1* and *TXNRD1* are known for their ROS buffering capacities and their ability to promote protein homeostasis (Heinze et al., 2018). *PRDX1* and *TXNRD1* are targets of the transcription factor erythroid2-related factor 2 NFE2L2, which regulates the transcription of cytoprotective factors, and activation of NFE2L2 correlates with life expectancy (Malhotra et al., 2010; Lewis et al., 2015). Another mechanism could be the unique split-ribosome structure and accuracy of these ribosomes, which significantly improve the translational fidelity in these animals. This mechanism might, despite higher protein damage levels, prevent supersaturation of aggregation-prone proteins in naked mole-rats (Azpurua et al., 2013).

Just like the proteome of *A. islandica*, the proteomes of naked mole-rats, Mexican free-tailed bats and cave myotis bats are very resistant to unfolding stressors like urea (Pérez et al., 2009; Salmon et al., 2009). In naked mole-rats, however, increased chaperone levels do seem to play a role in the protection of the proteome against unfolding stressors. ATP-dependent chaperone levels were elevated under normal and heat shock conditions in cultured fibroblasts from naked mole-rats compared with cells cultured from short-living counterparts (Rodriguez et al., 2014; Pride et al., 2015). These elevated chaperone levels were also observed in cells from other long-living animal species, including sugar gliders, the Australian black flying fox and the cave nectar bat (Rodriguez et al., 2014, 2016; Pride et al., 2015; Chionh et al., 2019).

The importance of protein degradation for protein homeostasis is reflected in the elevated macroautophagy rate and proteasomal activity in the improved protein homeostasis network of naked mole-rats (Pérez et al., 2009; Rodriguez et al., 2014, 2016; Triplett et al., 2015). Particularly interesting is the stress-resistance of naked mole-rat proteasomes compared with those of other species. Proteasomes of naked mole-rats retained their activity after treatment with increasing concentrations of different proteasome competitive inhibitors, while mouse proteasomes lost all activity after exposure to low concentrations of the same inhibitors (Rodriguez et al., 2014). Interestingly, the proteasomes of naked mole-rats lost their resistance to proteasomal inhibitors when resuspended in proteasome-depleted mouse cytosolic extracts (Rodriguez et al., 2014). Conversely, enhanced resistance and increased levels of proteasomal activity were observed for mouse, yeast, and human proteasomes that were resuspended in cytosolic extracts of naked mole-rats. This indicates that factors specifically present in the cytosol of naked mole-rats are responsible for the improved proteolytic resistance and activity. Although the exact composition of this cytosolic factor remains unknown, inhibition of Hsp72 and its co-chaperone Hsp40 reduced the activity of proteasomes, indicating that these factors contribute to resistance to proteasomal stressors in naked mole rats (Rodriguez et al., 2014). This is particularly interesting as no Hsp has previously been described to specifically promote proteasome activity and to protect proteasomes from proteasome-specific inhibitors. The contribution of an active proteolytic system to healthy aging has also been proposed in humans. The expression of proteasomal components is reduced in aged individuals, whereas expression in centenarians was found to be similar to the expression levels in much younger individuals (Chondrogianni et al., 2000). Increased levels of proteasomal subunits could contribute to a more efficient degradation of (oxidized) proteins and therefore to a longer and healthier life.

An improved protein homeostasis in long-living animal species does not seem to depend on a single pathway, but rather on a combination of multiple optimized pathways (Figure 3). Each animal species has its own combination of mechanisms that protect its proteome from aging. The proteomes of long-living animal species have been optimized for living under specific environmental conditions. For example, reduced ROS production seems beneficial, but not all animals can regulate this. Bats and birds have to cope with high metabolic rates during flight (Munshi-South and Wilkinson, 2010), and naked mole-rats might have to deal with high concentrations of heavy metals in the soil they are living in (Rodriguez et al., 2016). Whereas some bats and the bivalve mollusk *A. islandica* might have optimized their DNA repair mechanisms and/or antioxidant levels to prevent protein damage (Brunet-Rossinni, 2004; Podlutsky et al., 2005; Wilhelm Filho et al., 2007; Ungvari et al., 2013; Huang et al., 2020), naked mole-rats have improved their refolding and degradation capacities to deal with damaged proteins (Pérez et al., 2009; Rodriguez et al., 2014, 2016; Pride et al., 2015; Triplett et al., 2015). Some animal species have evolved unique mechanisms to prolong their health and lifespan, as for example the cytosolic factor that promotes proteasome activity and resistance to proteasomal stressors in naked mole-rats (Rodriguez et al., 2014). Long-living species generally seem to have evolved an improved protein structure, strong DNA repair mechanisms, and more stable proteomes. Multiple studies indicate that additional, yet unknown mechanisms may increase protein homeostasis and longevity. For example, better resistance to protein unfolding could be explained by unknown chaperones that prevent protein unfolding or the existence of more stable protein conformations (Pérez et al., 2009; Salmon et al., 2009). Animal models that are capable of regeneration like the flatworm *Macrostomum lignano* or immortal cell lines might also extend our knowledge about the role of protein homeostasis in longevity (Vilchez et al., 2012a; Noormohammadi et al., 2016; Mouton et al., 2018). Recent studies have revealed that genes that are associated with increased longevity in other organisms are naturally upregulated with age in the long-living, regeneration-capable flatworm *M. lignano* (Mouton et al., 2018). Further identification of genes that are upregulated in this animal model and their role in protein homeostasis pathways could contribute to our knowledge about how protein homeostasis affects aging. In addition, enhanced expression of the CCT8 subunit of the chaperonin TRiC/CCT complex was shown to play an important role in proteome stability in human pluripotent stem cells. Upon differentiation, CCT8 levels decrease and differentiated cells become more susceptible to a decline in protein homeostasis and aging (Noormohammadi et al., 2016).

## Conclusion

To prevent or delay age-related diseases, we need to understand the underlying mechanisms that are involved in aging and disease. Understanding the differences in protein homeostasis between closely related animal species with different lifespans is a useful way of acquiring knowledge about the mechanisms of aging. A healthy, long life is clearly not dependent on the optimization of a single pathway. The multiple adaptations in the naked mole-rat which increase its resistance to cancer and neurodegenerative diseases are a clear example of this. The use of naturally long-living animal species like bivalve mollusks, bats, and naked mole-rats may also uncover ways to improve health and prolong lifespan. Further research of long-living animal models may therefore contribute to uncover the pathways that are involved in preventing amyloid formation and toxic sequestration of aggregation-prone proteins. These studies might help us understand these pathways and allow us to develop strategies to suppress age-related protein toxicity.

## Author Contributions

AP wrote the manuscript with the contribution and input from EN and the reviewers. Both authors contributed to the article and approved the submitted version.

## Conflict of Interest

The authors declare that the research was conducted in the absence of any commercial or financial relationships that could be construed as a potential conflict of interest.
